# HBV DNA and HBsAg: Early Prediction of Response to Peginterferon α-2a in HBeAg-Negative Chronic Hepatitis B

**DOI:** 10.7150/ijms.39775

**Published:** 2020-02-04

**Authors:** Chong Zhang, Zhengrong Yang, Ziyi Wang, Xiaoguang Dou, Qiuju Sheng, Yanwei Li, Chao Han, Yang Ding

**Affiliations:** Department of Infectious Diseases, Shengjing Hospital of China Medical University, Shenyang, 110022, China

**Keywords:** chronic hepatitis B, HBeAg, prospective study

## Abstract

**Objective**: The proportion of hepatitis e antigen (HBeAg)-negative chronic hepatitis B (CHB) patients in China has increased rapidly. However, the response of these patients to peginterferon (peg-IFN) treatment is poor, and the antiviral treatment strategies are inconsistent. This study aimed to investigate the role of hepatitis B virus (HBV) DNA and hepatitis B surface antigen (HBsAg) in early prediction of response in HBeAg-negative CHB patients receiving peg-IFN α-2a.

**Patients and Methods**: Treatment-naïve HBeAg-negative patients were involved in this prospective study during 2014-2018. The HBV DNA and HBsAg were quantified at baseline and during treatment (weeks 12, 24 and 48) in sera. The factors associated with HBV DNA undetectable and HBsAg <100 IU/ml at treatment 48 weeks were assessed.

**Results**: This study involved 45 patients. There was HBV DNA undetectable in 36 cases (80%), including 19 (52.8%) with HBsAg <100 IU/ml at week 48. The HBV DNA <2.0 log_10_IU/ml at week 24 (PPV = 96.9%, NPV = 66.7%, *P* = 0.018) was an independent predictor of HBV DNA undetectable at week 48. The HBsAg <800 IU/ml at baseline (PPV = 92.1%, NPV = 69.7%, *P* = 0.054) and HBsAg decline >5.00-fold at week 24 (PPV = 83.3%, NPV = 77.8%, *P* = 0.038) were independent predictors of HBsAg <100 IU/ml and HBV DNA undetectable at week 48.

**Conclusion**: Early on-treatment quantification of HBV DNA and HBsAg in patients with HBeAg-negative CHB treated with peg-IFN α-2a may help identify those likely to be cured by this method and optimize therapy strategies.

## Introduction

Chronic hepatitis B virus (HBV) infection is a health problem affecting over 240 million people worldwide 1. Nearly 40% of those with chronic HBV infection who do not receive antiviral therapy will deteriorate into cirrhosis or hepatocellular carcinoma (HCC), with a high risk of liver function decompensation 2. The incidence of hepatitis e antigen (HBeAg)-negative chronic hepatitis B (CHB) in China has increased rapidly in recent years 3. It represents a late phase in the course of the infection, accompanied by much more serious fibrosis and easily deteriorates in comparison with HBeAg-positive CHB 4. Thus, HBeAg-negative patients need much more active treatment.

Guidelines worldwide currently recommend peginterferon (peg-IFN), entecavir and tenofovir as the first-line treatment for CHB 5-7. Compared with nucleos(t)ide analogs, peg-IFN has advantages such as shorter therapy course, lower drug resistance rate and an effect of immunomodulatory function. Therefore, peg-IFN is preferred for HBeAg-negative patients.

In the last decade, much research has focused on optimizing CHB therapy strategies. Antiviral therapy is expected to achieve long-term good prognosis through short-term treatment and it is important to explore associative factors used as prognostic prediction. It was shown that HBV DNA inhibition and clearance and low levels of hepatitis B s antigen (HBsAg) are important factors in delaying disease progression 8-11. Notably, HBsAg quantitative level plays an important role in predicting the efficacy of antiviral therapy and the long-term prognosis of the disease. Several studies have suggested that HBsAg level is an independent predictor for progression of CHB, and a low level of HBsAg (<100 IU/ml) is followed by reduced risk of disease deterioration 11-13.

However, only a small portion of HBeAg-negative patients achieve sustained response when treated with peg-IFN. It is therefore a major challenge to identify the patients likely to benefit from peg-IFN therapy as early as possible in the treatment course. Several recent studies suggested that age and gender of patients, HBV genotype and dynamics of HBsAg and HBV DNA in the treatment course can be used to predict the response to interferon-based therapy 14,15. However, most of these studies were registered randomized controlled trials, and the data were mostly from Caucasians and African Americans. Our study is a prospective, real-world, observational study aimed at HBeAg-negative CHB patients from China to determine the role of early on-treatment quantitative HBV DNA and HBsAg in prediction of response of patients treated with peg-IFN α-2a.

## Patients and Methods

### Patients

Treatment-naïve HBeAg-negative CHB patients were involved in this study during 2014-2018. The patients were from China and aged 18-65 years. Patients eligible for this study had been positive for HBsAg for >6 months, were HBeAg-negative, had a serum HBV DNA level of ≥2000 IU/ml, had repeated elevated serum alanine aminotransferase (ALT) levels [>2 but ≤10 times the upper limit of the normal range (ULN)] and had serum total bilirubin level ≤2 ULN 5. Exclusion criteria were as follows: history of anti-HBV treatment; coinfection with hepatitis C virus, hepatitis D virus or human immunodeficiency virus; other acute or chronic liver diseases or decompensated liver disease; pregnancy; mental illness, thyroid disease, autoimmune disease, retinal disease, serious infection and severe acute or chronic cardiopulmonary insufficiency.

All patients received 180 μg of peg-IFN α-2a weekly, with duration of 48 weeks followed up every 4 weeks. A decision whether to continue treatment or change strategy after 48 weeks was made according to the response during therapy. For patients whose HBV DNA has dropped to the lower limit of detection and HBsAg has dropped to 10 IU/mL at 48 weeks of treatment with Peg-IFN, the treatment can be extended to 72 weeks or longer to pursue clinical cure. Otherwise, it is recommended to stop Peg-IFN and treat with nucleoside analogs for the long term 16.

All patients gave written informed consent. This study was approved by The Ethical Committees of Shengjing Hospital.

### Laboratory measurements

The HBV viral load and HBsAg and ALT levels of patients were determined at the baseline and during treatment (weeks 12, 24 and 48) in the sera. Serum HBV DNA was measured by Roche COBAS AmpliPrep/COBAS TaqMan HBV Quantitative Test, Version 2.0 (Roche Diagnostics, Indianapolis, IN), with dynamic range 20-1.7 E8 IU/ml. The HBsAg level was measured by Abbott Architect Plus i2000 analyzer (Abbott, Abbott Park, IL), with a lower limit of detection of 0.05 IU/ml. The ALT level was determined by Beckman AU5800 automatic biochemical analyzer (Beckman Coulter Inc., Atlanta, GA).

### Statistical analysis

Statistical software SPSS 21.0 (SPSS, Chicago, IL) was used for statistical processing and analysis. Continuous variables were analyzed for normal distribution using the Kolmogorov-Smirnov test. Normally distributed data are presented as mean ± standard deviation (SD) and their inter-group comparison was performed with independent sample *t* test or *t′* test; the non-normally distributed data are presented as median (P250, P750) and their inter-group comparison was performed with the Mann-Whitney U test. Categorical variables are presented as number and percentage and their inter-group comparison was performed with *χ*^2^ test. Significant differences required* P* < 0.05. The predictive analysis of results was performed using the receiver operating characteristic curve (ROC) and logistic regression, with *P* < 0.1 indicating a significant difference.

## Results

### Baseline characteristics

During the observation period, a total of 45 patients completed 48 weeks of peg-IFN α-2a therapy, of whom 33 were male (73.3%). The mean age was 41.47 ± 10.43 (mean ± SD) years. At the baseline, serum HBV viral load was 5.33 log_10_ IU/ml (SD: 1.45 log_10_ IU/ml, range: 3.38-7.78 log_10_ IU/ml), and serum HBsAg level was 3.15 log_10_ IU/ml (SD: 0.61 log_10_ IU/ml, range: 1.38-4.35 log_10_ IU/ml).

### Factors associated with HBV DNA undetectable at treatment week 48

After 48 weeks of peg-IFN α-2a treatment, HBV viral loads were undetectable in 36 cases, making an undetectable rate of 80%. Serum HBV viral loads at baseline, weeks 12 and 24 of patients with HBV DNA undetectable at week 48 were significantly lower than those with HBV DNA detectable at week 48 {5.03±1.29 (mean ± SD) log_10_ IU/ml, *P* = 0.004; 1.61 ± 1.34 (mean ±SD) log_10_ IU/ml, *P*<0.001; 0 (0, 1.54) [median (interquartile range)] log_10_ IU/ml, *P* < 0.001; respectively}. There was a significantly lower HBsAg level at week 24 in HBV DNA undetectable patients than in HBV DNA detectable patients (2.19 ± 1.20 vs 3.20 ± 0.54 log_10_ IU/ml, *P* = 0.017). Furthermore, although non-significant, HBV DNA undetectable cases had much lower HBsAg levels at both baseline and week 12, and much higher HBsAg decline at week 24 from the baseline (7.59-fold vs 2.00-fold, *P* = 0.055), compared with HBV DNA detectable cases (Table 1).

### Prediction of HBV DNA undetectable at treatment week 48

**Figure 1:**
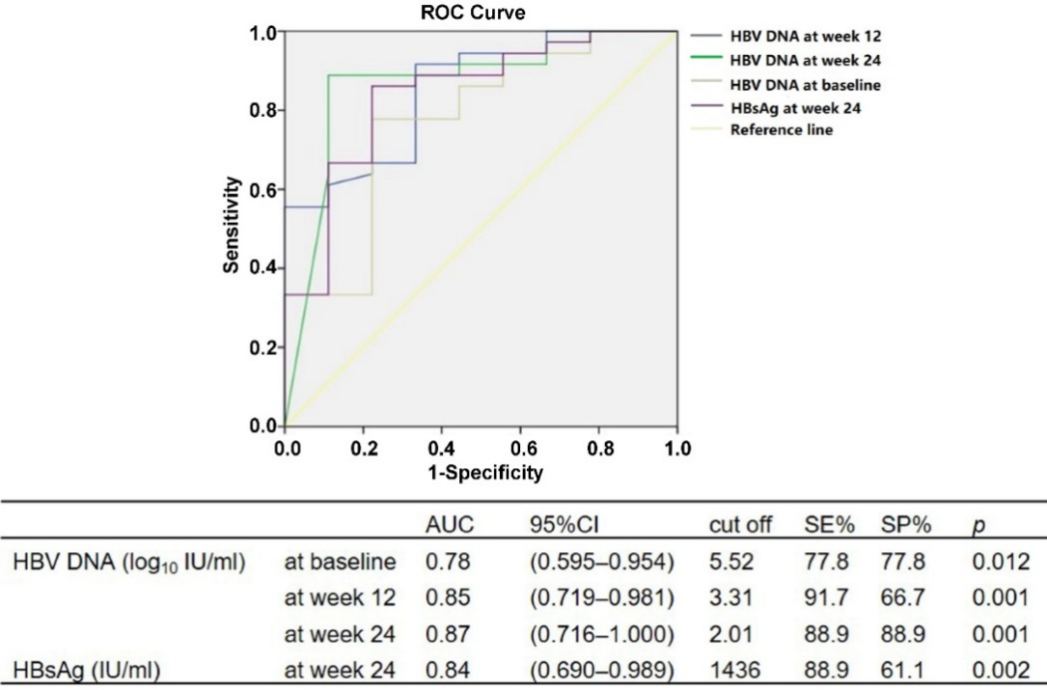
ROC analysis for HBV DNA level at baseline and weeks 12 and 24, and HBsAg at week 24, for prediction of HBV DNA undetectable at week 48 during peg-IFN α-2a therapy.

Using ROC, we analyzed the cutoff values of HBV DNA and HBsAg levels during baseline and weeks 12 and 24 of therapy to predict HBV DNA undetectable at week 48. The serum HBV DNA of <5.52 log10 IU/ml at baseline (*P* = 0.012), <3.31 log_10_ IU/ml at week 12 (*P* = 0.001) and <2.01 log_10_ IU/ml at week 24 (*P* = 0.001), and serum HBsAg level <1436 IU/ml at week 24 (*P* = 0.002), could predict HBV DNA undetectable after 48 weeks of peg-IFN α-2a therapy (Fig. 1).

The approximate predictive values for the critical values of various predictive indicators in the ROC analysis were used to conduct univariate and multivariate analyses of binary logistic regression. The serum HBV DNA of <5.5 log_10_ IU/ml at baseline (*P* = 0.005), <3.3 log_10_ IU/ml at week 12 (*P* = 0.001) and <2.0 log_10_ IU/ml at week 24 (*P* < 0.001), and serum HBsAg level <1400 IU/ml at week 24 (*P* = 0.010), could predict HBV DNA undetectable after 48 weeks of peg-IFN α-2a treatment. The HBV DNA of <2.0 log_10_ IU/ml at week 24 was an independent predictor for HBV DNA undetectable at week 48, with positive predictive value (PPV) of 96.9% and negative predictive value (NPV) of 66.7% (OR = 29.7, *P* = 0.018).

### Factors associated with HBsAg <100 IU/ml at treatment week 48

In 36 cases of HBV DNA undetectable after 48 weeks of peg-IFN α-2a treatment, serum HBsAg levels in 19 cases (52.8%) were <100 IU/ml. Compared with patients with HBsAg ≥100 IU/ml at treatment week 48, HBsAg levels in patients with HBsAg <100 IU/ml were significantly lower at baseline and weeks 12 and 24 [2.79 ± 0.65 vs 3.37 ± 0.38 (mean ± SD) log_10_ IU/ml, *P* = 0.003; 2.17 ± 1.11 vs 3.15 ± 0.37 (mean ± SD) log_10_ IU/ml, *P* = 0.001; 1.51 ± 1.28 vs 2.93 ± 0.38 (mean ± SD) log10 IU/ml, *P* < 0.001; respectively]. Furthermore, HBsAg declines from baseline after 12 and 24 weeks of treatment were significantly higher in patients with HBsAg <100 IU/ml at week 48 compared with those HBsAg ≥100 IU/ml {0.31 (0.14, 0.99) vs 0.12 (0.01, 0.25) [median (interquartile range)] log_10_ IU/ml, *P* = 0.042, for week 12; 1.28 ± 0.94 vs 0.43 ± 0.40 (mean ± SD) log_10_ IU/ml, *P* = 0.001, for week 24; respectively} (Table 2).

### Prediction of HBsAg <100 IU/ml at treatment week 48

**Figure 2:**
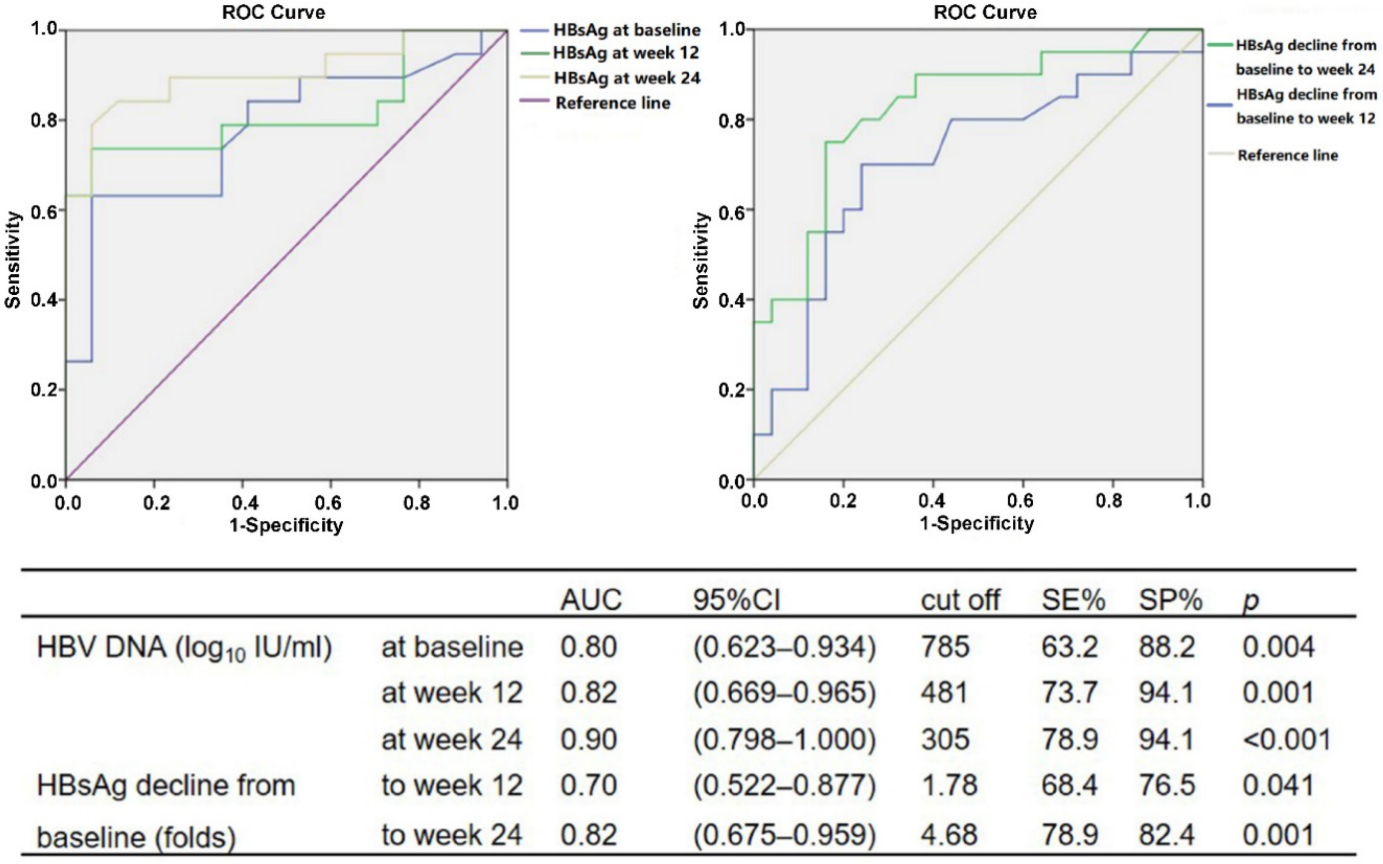
ROC analysis for HBsAg level at baseline and weeks 12 and 24, and HBsAg decline from baseline to weeks 12 and 24, for prediction of HBsAg < 100 IU/ml in patients with HBV DNA undetectable at week 48 during peg-IFN α-2a therapy.

The cutoff values of HBV DNA and HBsAg levels during baseline and weeks 12 and 24 of therapy to predict HBsAg < 100 IU/ml at week 48 were analyzed using ROC. The serum HBsAg of <785 IU/ml at baseline (*P* = 0.004), <481 IU/ml at week 12 (*P* = 0.001) and <305 IU/ml at week 24 (*P* < 0.001) could be used to predict HBsAg <100 IU/ml in patients with HBV DNA undetectable after 48 weeks of peg-IFN α-2a therapy. Similarly, HBsAg declines of >1.78-fold from baseline to week 12 (*P* = 0.041) and >4.68-fold to week 24 (*P* = 0.001) compared with baseline could also be used as predictors (Fig. 2).

Using the results of ROC analysis, the approximate predictive values for the critical values of various predictive indicators were used to conduct univariate and multivariate analyses of binary logistic regression. The serum HBsAg levels <800 IU/ml at baseline (*P* = 0.004), <500 IU/ml at week 12 (*P* < 0.001) and <300 IU/ml at week 24 (*P* < 0.001), together with HBsAg decline >2.00-fold from baseline to week 12 (*P* = 0.048) and >5.00-fold to week 24 (*P* = 0.001) could predict HBsAg <100 IU/ml in patients with HBV DNA undetectable after 48 weeks of peg-IFN α-2a treatment. Among them, HBsAg <800 IU/ml (PPV = 92.1%, NPV = 69.7%, OR = 29.56, *P* = 0.054) and HBsAg decline of >5.00-fold to week 24 (PPV = 83.3%, NPV = 77.8%, OR = 43.16, *P* = 0.038) were independent predictors for HBsAg <100 IU/ml at week 48; meanwhile, HBV DNA was undetectable.

## Discussion

Peginterferon as a first-line drug for CHB has been widely used in treatment of HBeAg-positive and -negative patients. For HBeAg-negative patients, the Chinese guidelines recommend the adjustment of treatment strategy based on HBsAg decline from baseline 5; however, HBeAg-negative CHB patients tend to have a lower baseline level of HBsAg. This may lead to missing the opportunity to continue peg-IFN therapy for some patients, if the clinical medication regimen is only adjusted according to HBsAg decline. Consequently, we not only considered the HBsAg declines but also their absolute values in our research, with the aim to provide new solutions for antiviral therapy in HBeAg-negative patients.

Many studies have suggested that inhibition and clearance of HBV DNA and a low level of HBsAg are the important factors delaying disease progression and the accumulative incidence rate of HCC is positively correlated with serum HBV DNA level 8-11. HBsAg level is an independent predictive factor for progression of CHB, and a lower level of HBsAg (<100 IU/ml) can decrease the risk of disease deterioration 11,17,18. Additionally, the recommended treatment course of peg-IFN is 48 weeks in the Chinese guidelines 5. Thus, the relevant monitoring period in this study was up to treatment week 48, when HBV DNA was undetectable and HBsAg <100 IU/ml.

Many studies have shown that serum HBV DNA and HBsAg levels at treatment weeks 12 and 24 have higher predictive values for viral response 19-21. Therefore, we evaluated the effects of serum levels of HBV DNA and HBsAg at baseline and weeks 12 and 24 on prediction of HBV DNA undetectable and HBsAg <100 IU/ml after therapy for 48 weeks. Reducing the time of detection can greatly reduce the economic and living burden for patients.

In the study by Peng *et al.*, HBV DNA levels at baseline and at treatment weeks 12 and 24 were independent predictors for viral response at week 48, the cut-offs for which were 4.3 (OR = 7, *P* = 0.02), 3.0 (OR = 7.9, *P* = 0.02) and 2.5 log_10_ copies/ml (OR = 22.3, *P* = 0.008), respectively 22. Another large-scale clinical study demonstrated that lower HBV viral load and higher ALT levels at baseline were important factors for acquiring SVR at follow-up week 24 after peg-IFN treatment 23. In the present study, serum HBV DNA of <5.5 log_10_ IU/ml at baseline, <3.3 log_10_ IU/ml at week 12 and <2.0 log_10_ IU/ml at week 24, and serum HBsAg <1400 IU/ml at week 24, could predict HBV DNA undetectable at week 48 of peg-IFN α-2a treatment. This suggests that detection of HBV DNA or HBsAg during treatment is critical for predicting the negative conversion of HBV DNA at 48 weeks. Notably, HBV DNA <2 log_10_ IU/ml at week 24 was an independent predictor with a greater level of significance.

We found that HBsAg <800 IU/ml at baseline, <500 IU/ml at week 12 and <300 IU/ml at week 24, together with HBsAg decline of >2.00-fold from baseline to week 12 and >5.00-fold to week 24, could predict HBsAg <100 IU/ml at week 48 of peg-IFN α-2a treatment. This indicates that HBsAg and its decline from the baseline at each test time during treatment are important for predicting low HBsAg levels at week 48. In particular, baseline HBsAg levels and 24-week HBsAg decline are significant. Briefly, for an HBeAg-negative CHB patient treated with peg-IFN, it was only necessary to detect HBsAg levels at baseline and week 24 of treatment. The level of baseline <800 IU/ml means that the patient has a 92.1% probability of HBsAg <100 IU/ml at treatment week 48; while HBsAg decline at week 24 by >5.00-fold from baseline means that the patient has an 83.3% probability of HBsAg <100 IU/ml at week 48 of treatment.

For HBeAg-negative CHB, the current guidelines recommend the use of log values of HBV DNA and HBsAg decline to predict the effect of peg-IFN therapy. However, most of the research population comprises Europeans and Americans. In this study, the subjects were a Chinese population with genotypes B and C, and quantitative HBV DNA and HBsAg were predictors. We analyzed the prediction of HBV DNA undetectable and HBsAg <100 IU/ml at week 48 and obtained the critical values of these indicators. The limitation is that this study is single-centered and of small sample size. Currently, some patients are receiving the treatment and follow-up in the clinic of our hospital, and a subsequent study with a larger sample size will be conducted. Because only four patients demonstrated the negative conversion of HBsAg at treatment week 48, the prediction of HBsAg clearance was not analyzed. At the end of the treatment, nine patients with HBsAg <10 IU/ml were still under the follow-up for the prolonged course of treatment, and efficacy will be further observed.

## Conclusion

In conclusion, for HBeAg-negative CHB patients treated with peg-IFN α-2a, baseline is the first checkpoint to understand their initial treatment values. Based on whether the baseline HBsAg is <800 IU/ml, a preliminary prediction is made on whether HBsAg would be <100 IU/ml at 48 weeks of treatment. The second checkpoint to predict is at treatment week 24, at which HBV DNA <2.0 log_10_ IU/ml can be used to predict whether HBV DNA is negative at week 48, and reduction of HBsAg >5.00-fold from baseline can be used to predict whether HBsAg is <100 IU/ml at week 48.

## Figures and Tables

**Table 1:** Characteristics of HBV DNA undetectable and detectable patients at treatment week 48.

Characteristics	HBV DNA undetectable (*n* = 36)	HBV DNA detectable (*n* = 9)	*P*
Age (years)	41.78 ± 9.88	40.22 ± 13.01	0.694
Sex (male/female)	27/9	6/3	0.682
Baseline HBsAg (log_10_ IU/ml)	3.07 ± 0.61	3.51 ± 0.53	0.051
HBV DNA (log_10_ IU/ml)	5.03 ± 1.29	6.65 ± 1.49	0.004
Week 12 HBsAg (log_10_ IU/ml)	2.63 ± 0.97	3.25 ± 0.53	0.075
HBsAg decline (log_10_ IU/ml)^†^	0.23 (0.05, 0.68)	0.11 (-0.07, 0.57)	0.348
HBV DNA (log_10_ IU/ml)	1.61 ± 1.34	3.78 ± 1.54	<0.001
HBV DNA decline (log_10_ IU/ml)	3.42 ± 1.10	2.78 ± 1.30	0.136
Week 24 HBsAg (log_10_ IU/ml)	2.19 ± 1.20	3.20 ± 0.54	0.017
HBsAg decline (log_10_ IU/ml)	0.88 ± 0.85	0.30 ± 0.42	0.055
HBV DNA (log_10_ IU/ml)^ †^	0 (0, 1.54)	2.55 (2.05, 4.06)	<0.001
HBV DNA decline (log_10_ IU/ml)	4.30 ± 1.35	3.53 ± 1.54	0.241

HBV, hepatitis B virus; HBsAg, hepatitis B surface antigen. †Expressed as median (interquartile range). Other continuous variables expressed as mean ± SD

**Table 2:** Characteristics of patients with HBsAg <100 IU/ml and ≥100 IU/ml at treatment week 48.

Characteristics	HBsAg <100 IU/ml (*n* = 19)	HBsAg ≥100 IU/ml (*n* = 17)	*P*
Age (years)	40.68 ± 10.43	43.00 ± 9.38	0.491
Sex (male/female)	15/4	12/5	0.423
Baseline HBsAg (log_10_ IU/ml)	2.79 ± 0.65	3.37 ± 0.38	0.003
HBV DNA (log_10_ IU/ml)	4.84 ± 1.26	5.24 ± 1.34	0.358
Week 12 HBsAg (log_10_ IU/ml)	2.17 ± 1.11	3.15 ± 0.37	0.001
HBsAg decline (log_10_ IU/ml)^†^	0.31 (0.14, 0.99)	0.12 (0.01, 0.25)	0.042
HBV DNA (log_10_ IU/ml)	1.33 ± 1.20	1.92 ± 1.45	0.187
HBV DNA decline (log_10_ IU/ml)	3.51 ± 0.89	3.32 ± 1.31	0.606
Week 24 HBsAg (log_10_ IU/ml)	1.51 ± 1.28	2.93 ± 0.38	<0.001
HBsAg decline (log_10_ IU/ml)	1.28 ± 0.94	0.43 ± 0.40	0.001
HBV DNA (log_10_ IU/ml)^ †^	0 (0, 1.31)	0 (0, 1.91)	0.219
HBV DNA decline (log_10_ IU/ml)	4.35 ± 1.13	4.23 ± 1.62	0.784

HBV, hepatitis B virus; HBsAg, hepatitis B surface antigen. †Expressed as median (interquartile range). Other continuous variables expressed as mean ± SD

## Abbreviations

HBV: hepatitis B virus
HCC: hepatocellular carcinoma
HBeAg: hepatitis e antigen
CHB: chronic hepatitis B
peg-IFN: peginterferon
HBsAg: hepatitis B s antigen
ALT: alanine aminotransferase
ULN: upper limit of the normal range
SD: standard deviation
ROC: receiver operating characteristic curve
PPV: positive predictive value
NPV: negative predictive value
SVR: sustained viral response
